# Dyshidrotic Eczema: A Common Cause of Palmar Dermatitis

**DOI:** 10.7759/cureus.10839

**Published:** 2020-10-07

**Authors:** Paola Michelle Calle Sarmiento, Juan Jose Chango Azanza

**Affiliations:** 1 Internal Medicine, Catholic University of Cuenca, Cuenca, ECU; 2 Internal Medicine, University of Connecticut School of Medicine, Farmington, USA

**Keywords:** dyshidrotic eczema, pompholyx, acute palmoplantar eczema

## Abstract

Dyshidrotic eczema (DE) or acute palmoplantar eczema is a common cause of hand and foot dermatitis in adults. It is a recurrent vesicular eruption affecting the soles, palms, or both. It is very pruriginous and generally appears suddenly. It creates vesicles that, on physical examination, can look similar to "tapioca pudding", which is the characteristic clinical feature of this disorder. It is more common in young adults and affects men and women equally. In this report, we present the case of a 56-year-old man with no relevant past medical history who presented to the hospital with vesicular lesions in his hands and maculopapular lesions in his arms and legs. The patient had characteristic lesions in his right hand consistent with DE and negative workup for bullous pemphigoid, scabies, and bacterial, fungal, and viral infections.

## Introduction

Dyshidrotic eczema (DE) or acute palmoplantar eczema is a common cause of hand dermatitis in adults. It accounts for 5-20% of the causes of DE [1]. It is a vesiculobullous disorder of the hands and soles. It is an intraepidermal spongiosis of the thick epidermis in which accumulation of edema causes the formation of small, tense, clear, fluid-filled vesicles on the lateral aspects of the fingers that can become large and form bullae [2]. The vesicles can have a deep-seated appearance, which is referred to as “tapioca pudding.” In severe cases, lesions can extend to the palmar area and affect the entire palmar aspect of the hand [2]. The diagnosis is mostly clinical and suggested by a recurrent rash of acute onset with vesicles and bullae located in the fingers extending to the palmar surfaces of the hands. We present a case of DE recognized clinically by the characteristic “tapioca pudding” appearance of the lesions located in the palmar aspect of the hand.

## Case presentation

**Figure 1:**
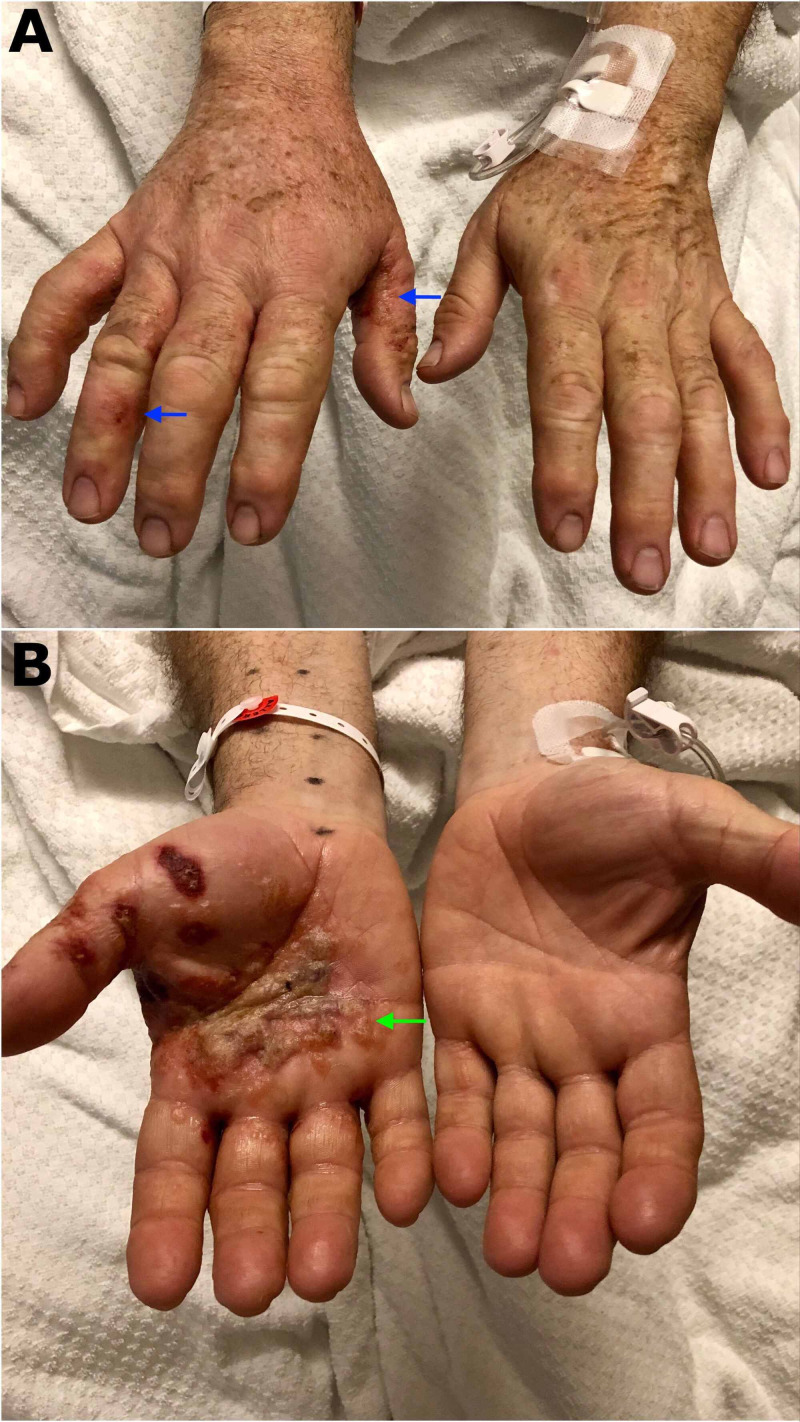
Vesicular lesions located in the lateral aspect of the fingers (blue arrows) (A). Palmar lesions containing vesicles and bullae that conglomerate getting the classic "tapioca pudding" appearance of dyshidrotic eczema (green arrow) (B)

**Figure 2:**
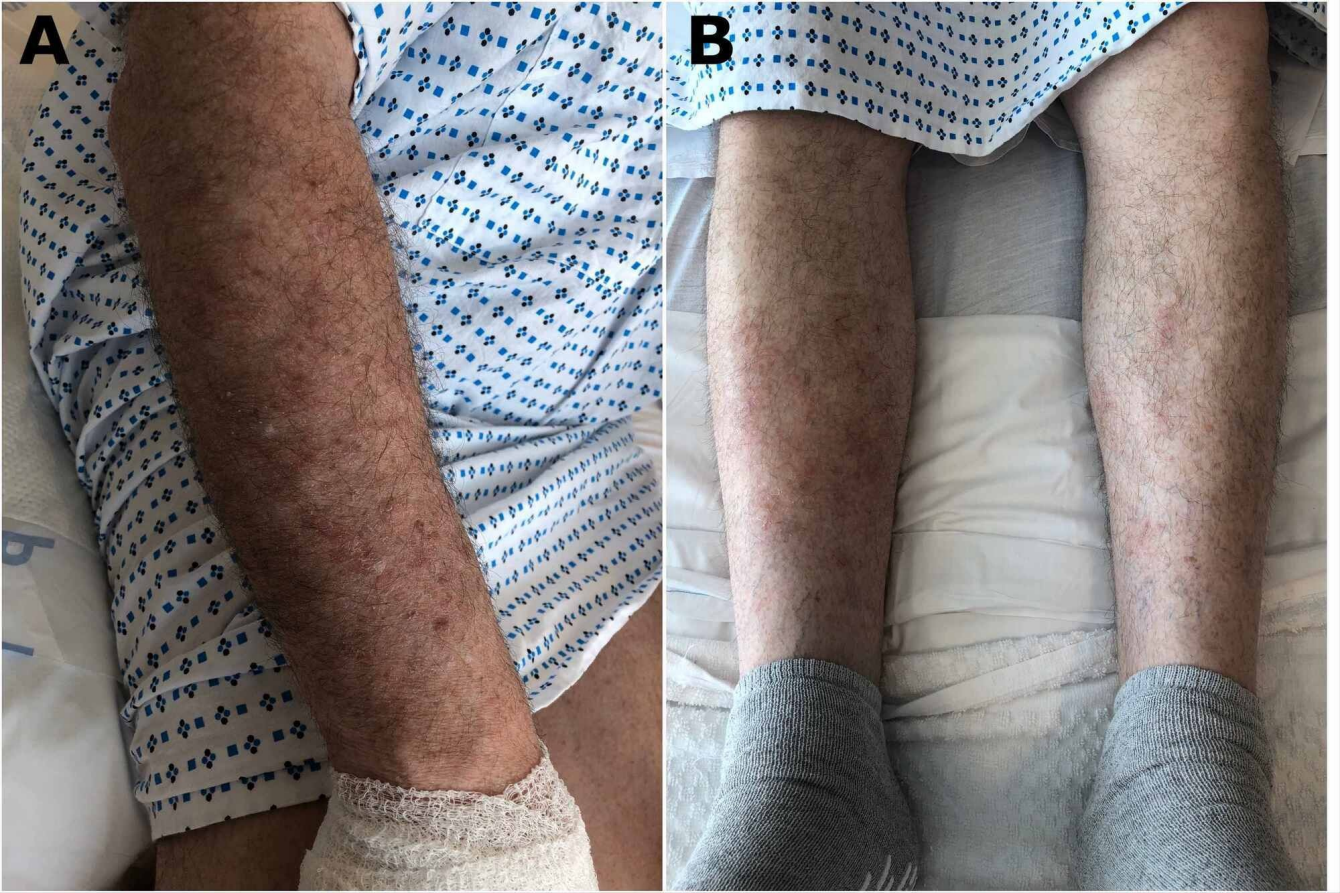
Maculopapular lesions with scratch marks located in the right forearm (A) and lower extremities (B)

A 56-year-old man with no relevant past medical history presented to the hospital due to a rash found in his hands, forearms, and legs. He had been in his usual state of health until a few days prior to his presentation when he had started developing intensely pruritic vesicular lesions in the lateral aspect of his bilateral fingers. He also developed a rash in his forearms and legs during the same period. He denied any other symptoms such as fevers, chills, fatigue, rhinorrhea, sore throat, neck pain, chest pain, shortness of breath, nausea or vomiting, abdominal pain, diarrhea, or urinary discomfort. He also denied any recent infections or sick contacts. He worked in an office as a manager. On presentation, his vital signs were within normal limits. Physical examination showed multiple vesicles and bullae located principally in the palmar aspect of the right hand with small vesicles in the lateral aspects of the left hand. The vesicles had a deep-seat appearance (“tapioca pudding”) (Figure 1). There were multiple maculopapular lesions with scratch marks located in the bilateral forearms and legs (Figure 2).

No other relevant physical examination findings were identified. He underwent a laboratory workup including complete blood count, chemistries, and inflammatory markers including erythrocyte sedimentation rate and C-reactive protein, which were all within normal limits. Further workup included a potassium hydroxide (KOH) preparation negative for fungal infection, and negative bacterial and viral cultures. A skin scraping with microscopy was performed, which was negative for scabies. Dermatology was consulted and agreed with a clinical diagnosis of DE and deemed that further workup including a skin biopsy was not necessary. The patient was treated with local care, which included the use of compresses and soaks with Burow’s solution. Drainage of the larger vesicles was also performed. Given the extensive lesions covering the bilateral hands, forearms, and legs, a short course of prednisone was recommended. Pruritus was treated effectively with diphenhydramine. The patient’s lesions improved significantly following the treatment.

## Discussion

DE is also known as acute palmoplantar eczema and is an intensely pruritic, vesicular eruption that presents in the hands, feet, or both. The term “dyshidrosis” was first used in 1873 to describe the blistering of the palms and soles, which was believed to be related to the sweat glands [3]. However, DE is not related to any abnormality of the sweat glands [2]. The exact prevalence of DE is unknown but it accounts for approximately 5-20% of the causes of hand eczema [1]. The exact cause of DE has not been established, but there are several risk factors for its occurrence, including atopic dermatitis, exposure to contact allergens and/or irritants, hyperhidrosis, smoking, exposure to ultraviolet light, and intravenous immunoglobulin use [4,5].

The clinical presentation of DE is a sudden eruption of intensely pruritic vesicles in the hands and feet, particularly in the lateral and dorsal aspects of the fingers [4]. The vesicles are deep-seated and can be multilocular with a characteristic “tapioca pudding” appearance and can coalesce into bullae. Therefore, the diagnosis of DE is mainly clinical. Secondary infection of the lesions can occur. A skin biopsy is rarely required and is usually indicated when there is a lack of improvement with treatment or if an infection is indicated in the differential diagnosis. Spongiosis is the main pathologic finding.

The management of DE focuses on the treatment of acute eruptions and long-term skincare. The treatment of acute DE is based on the severity of the presentation. Severity could be assessed by using the Dyshidrotic Eczema Area and Severity Index (DASI) score, which considers the number of vesicles, the severity of erythema, pruritus, and other characteristics. However, the DASI score is not applied broadly in medical practice [6]. In general, mild to moderate cases present with lesions that do not involve the entire palmar or plantar surfaces, have few crops of vesicles, erythema that is mild, pruritus that is not disabling, and no significant pain or discomfort [2]. Mild to moderate cases are treated with topical corticosteroids and calcineurin inhibitors. Topical tacrolimus and mometasone have also been used for treatment [7]. Severe disease is treated with systemic corticosteroids [3].

DE treatment is considered refractory when there is a lack of improvement after two to four weeks of adequate therapy. When the differential diagnosis is uncertain, then further workup for bacterial, fungal, and viral infections, skin patch testing, and skin biopsy can be considered [2]. Topical psoralen and ultraviolet A therapy (PUVA) can be used in refractory DE [8]. There might be a role for botulinum toxin use in DE for the management of cases with refractory pruritus [9].

## Conclusions

DE is a common cause of hand dermatitis. The identification of the condition by clinical features is crucial given that it is mainly a clinical diagnosis and biopsy is reserved for patients with refractory disease or suspected secondary infection. Learning how to identify the “tapioca pudding” appearance of the vesicular lesions in these disorders is important to differentiate it from other conditions.
